# The Cyclic Oligoadenylate Signaling Pathway of Type III CRISPR-Cas Systems

**DOI:** 10.3389/fmicb.2020.602789

**Published:** 2021-01-20

**Authors:** Fengtao Huang, Bin Zhu

**Affiliations:** Key Laboratory of Molecular Biophysics, the Ministry of Education, College of Life Science and Technology and Shenzhen College, Huazhong University of Science and Technology, Wuhan, China

**Keywords:** cyclic oligonucleotide, type III systems, CRISPR immune defense, CARF domain proteins, Cas10, CD-NTase

## Abstract

Type III CRISPR-Cas systems, which are widespread in both bacteria and archaea, provide immunity against DNA viruses and plasmids in a transcription-dependent manner. Since an unprecedented cyclic oligoadenylate (cOA) signaling pathway was discovered in type III systems in 2017, the cOA signaling has been extensively studied in recent 3 years, which has expanded our understanding of type III systems immune defense and also its counteraction by viruses. In this review, we summarized recent advances in cOA synthesis, cOA-activated effector protein, cOA signaling-mediated immunoprotection, and cOA signaling inhibition, and highlighted the crosstalk between cOA signaling and other cyclic oligonucleotide-mediated immunity discovered very recently.

## Introduction

CRISPR-Cas systems are known to provide adaptive immunity against viruses and plasmids in prokaryotes. Based on the composition of effector complexes, CRISPR-Cas systems were divided into two classes which could be further subdivided into six types (types I–VI) and multiple subtypes (Makarova et al., 2020b). Class 1 systems (including type I, III, and IV), which have multi-subunit effector complex, are widespread in bacteria and archaea; whereas class 2 (including type II, V, and VI), which contain single-subunit effector complex, are almost completely presented in bacteria (Mohanraju et al., 2016). The effector complexes of type I, II, and V (and possibly IV) target double-stranded DNA (dsDNA), while Type VI system targets RNA (Makarova et al., 2020b). Unlike them, type III effector complex targets both RNA and single-stranded DNA (ssDNA) of the invaders (Tamulaitis et al., 2017). The type III system can be further divided into six subtypes (III A–F), in which Type III-A/D system forms a Csm effector complex composed of five subunits (Csm 1–5) and a single CRISPR RNA (crRNA), while Type III-B/C forms a Cmr effector complex consisting of six subunits (Cmr 1–6) and a crRNA (Makarova et al., 2020b). The effector complexes of type III systems exhibit both target RNA cleavage activity and target RNA-activated ssDNA cleavage activity (Elmore et al., 2016; Estrella et al., 2016; Kazlauskiene et al., 2016). Type III systems provide immunity against invaders depending on the target RNA transcription (Deng et al., 2013; Goldberg et al., 2014). The crRNA-guided Csm/Cmr complexes recognize the complementary target RNA and cleave it into 6 nt nucleotide intervals using the multiple copies of Csm3 or Cmr4 subunit (Hale et al., 2009; Estrella et al., 2016; Kazlauskiene et al., 2016). Target RNA binding also activates the cyclic oligoadenylate (cOA) synthesis activity of Cas 10 subunit. More details about the transcription-dependent immunity and the structural basis of type III effector complexes and effector proteins had been reviewed elsewhere (Pyenson and Marraffini, 2017; Tamulaitis et al., 2017; Molina et al., 2020). In this review, we systematically discuss the recent advances in cOA signaling pathway of type III systems.

**Figure 1:**
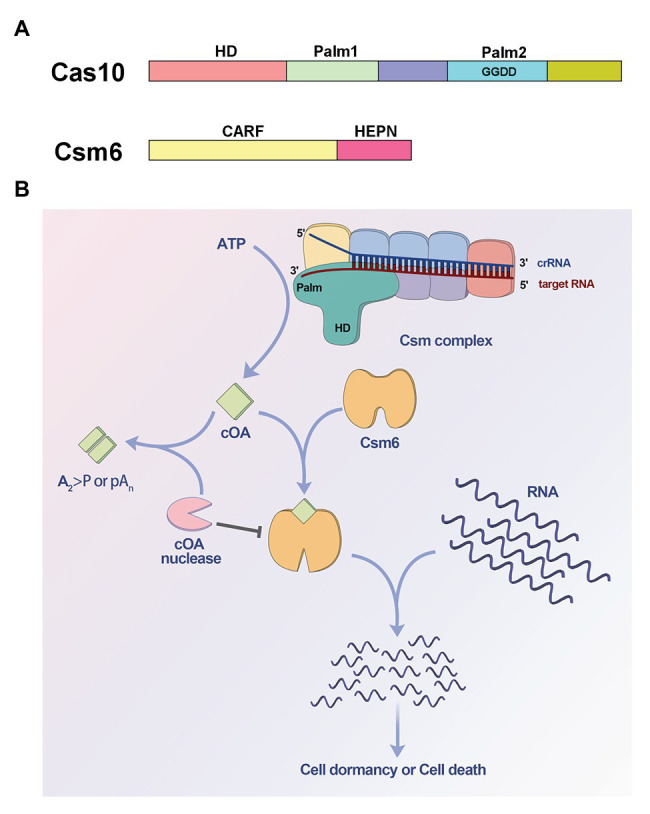
Cyclic oligoadenylate (cOA) signaling-mediated immunity in type III CRISPR-Cas systems. **(A)** Domain organization of Cas10 and Csm6. Cas10 contains an N-terminal histidine-aspartate (HD) domain and two Palm domains (Palm1 and Palm2), and the GGDD motif is inserted into Palm2 domain. Csm6 contains an N-terminal CRISPR-associated Rossman fold (CARF) domain and a C-terminal higher eukaryotes and prokaryotes nucleotide-binding (HEPN) domain. **(B)** Model for cOA signaling pathway of type III systems. The Palm domains of Cas10 subunit are activated and convert ATP into cOA molecules, when the target RNA is recognized by the CRISPR RNA (crRNA)-guided effector complex. The synthesized cOA allosterically activates Csm6 by binding the CARF domain, which subsequently degrades RNA non-specifically, resulting in host cell dormancy or cell death. On the other hand, cOA nucleases including ring nuclease and membrane-associated DHH-DHHA1 family nuclease can degrade cOA molecules to switch off cOA signaling, thereby acting as off-switch for the systems.

The Cas 10 subunit of type III effector complex and the ancillary ribonuclease Csm6/Csx1 are two important components involved in cOA signaling. Cas 10 contains an N-terminal histidine-aspartate (HD) domain and two Palm domains with a GGDD motif inserted into the second Palm domain (Tamulaitis et al., 2017; Figure 1A). The HD domain is responsible for ssDNA cleavage activity, while the Palm domains are homologous to nucleotide polymerases and nucleotide cyclase (Makarova et al., 2002, 2011; Zhu and Ye, 2012), and were hypothesized to synthesize cyclic nucleotides like cyclic di-AMP (Burroughs et al., 2015). However, there was no experimental evidence to verify the domains function for a long time (Koonin and Makarova, 2018). Csm6/Csx1 contains an N-terminal CRISPR-associated Rossman fold (CARF) domain which was predicted to sense nucleotide derivative and a C-terminal higher eukaryotes and prokaryotes nucleotide-binding (HEPN) domain which often functions as ribonuclease (Anantharaman et al., 2013; Makarova et al., 2014; Figure 1A). In 2017, two independent studies revealed that the two proteins were involved in a cOA signaling pathway, which had never been found in prokaryotes (Kazlauskiene et al., 2017; Niewoehner et al., 2017). It was found that the Palm domains were responsible for cOA synthesis, and the CARF domain of Csm6/Csx1 can sense the corresponding cOA (Kazlauskiene et al., 2017; Niewoehner et al., 2017; Figure 1B). When target RNA is recognized by effector complex, Cas10 subunit can be activated and can generate cOA, which in turn allosterically activates the ribonuclease Csm6/Csx1 through binding the CARF domain, resulting in non-specific RNA degradation (Kazlauskiene et al., 2017; Niewoehner et al., 2017; Figure 1B).

## Cas10 Activation-Triggered cOA Syxnthesis

Cas10 is the largest subunit of type III effector complex and is a signature protein of type III systems (Tamulaitis et al., 2017; Koonin and Makarova, 2018). The cOA synthesis activity of Cas10 is subject to tight spatial and temporal control. The Cas10 subunit is activated and converts ATP into cOA molecules only when target RNA is recognized by the crRNA-guided effector complex, and cOA synthesis will be deactivated abruptly following target RNA cleavage and dissociation from the effector complex (Kazlauskiene et al., 2017; Niewoehner et al., 2017; Rouillon et al., 2018). Unlike type I, II, and V CRISPR-Cas systems which distinguish self from non-self DNA in a protospacer adjacent motif (PAM)-dependent manner, type III systems were proposed to rely on the 5'-handle of crRNA (8 nt) and the 3'-flanking sequence of the target RNA to avoid autoimmunity (Marraffini and Sontheimer, 2010; Kazlauskiene et al., 2016; Tamulaitis et al., 2017). Non-complementarity between crRNA 5'-handle and 3'-flanking sequence of the target RNA is essential for Cas10 activation for cOA synthesis (Kazlauskiene et al., 2017). Previous studies showed type III systems were much tolerant of mismatches in target RNA (Pyenson et al., 2017; Goldberg et al., 2018; Rouillon et al., 2018). To some degree, target RNA binding-mediated Cas10 activation is also tolerant of crRNA-target mismatches, but base pairs in direct contact with Cas10 subunit, such as those adjacent to the 3' end of target RNA, are very stringent (Rouillon et al., 2018; Nasef et al., 2019). Base-pairing of 3'-flanking target RNA sequence to the 5'-handle of crRNA affects activation of both ssDNA cleavage and cOA synthesis (Guo et al., 2019; Johnson et al., 2019; Foster et al., 2020). Structural studies on Csm complexes show that the interaction between non-complementary 3'-flanking target RNA sequence and Cas10 subunit is crucial to induce a conformational change of Cas10 subunit for activation of its single-stranded DNase (ssDNase) and cOA synthetase activities (Jia et al., 2019c; You et al., 2019). Moreover, recent studies on the Cmr complex show that a unique stalk loop in Cmr3 is critical for avoiding autoimmunity and triggering Cas10 activation (Guo et al., 2019; Sofos et al., 2020). In addition, the length of the crRNA-target duplex also affects Cas10 activation. Twenty five base pairs or longer crRNA-target duplex are required for efficient activation of ssDNA cleavage and cOA generation (You et al., 2019; Sofos et al., 2020).

Since cOA synthesis was identified in Type III-A system of *Streptococcus thermophilus* and *Enterococcus italicus*, respectively (Kazlauskiene et al., 2017; Niewoehner et al., 2017), the Cas10 subunits of effector complexes from various bacteria and *archaea*, which harbor type III-A/B/D systems were verified to generate various cOA molecules (cOA_n_, *n* = 3–6; Han et al., 2018; Rouillon et al., 2018; Grüschow et al., 2019; Nasef et al., 2019; Foster et al., 2020). Notably, the major cOA species produced by effector complex is not always the one that activates the effector ribonuclease in the same system (Kazlauskiene et al., 2017; Rouillon et al., 2019; Smalakyte et al., 2020). That may be because the *in vitro* cOA synthesis could be affected by reaction conditions, thus the synthesized major cOA species may be different from those *in vivo* (Smalakyte et al., 2020). Recently, alternative nucleotide signal molecules were found to be synthesized by GDDEF cyclase, cGAS/DncV-like nucleotidyltransferases (CD-NTases), and ppGpp synthetase homolog (Hallberg et al., 2016; Ahmad et al., 2019; Whiteley et al., 2019), leading us to consider the existence of a subfamily of Cas10-like proteins that can synthesize other kinds of cyclic oligonucleotide molecules. Structure studies on Csm effector complexes bound to substrates (AMPPNP and ATP) have shown that each Palm domain has a conserved serine residue (Ser^273^ and Ser^549^ in the *Streptococcus thermophilus* Csm1), which forms hydrogen bonds with base of ATP and confers specificity for ATP (Jia et al., 2019a; You et al., 2019). Moreover, a biochemical study on Cmr effector complexes also shows that two conserved serine residues in the Palm 1 domain of Cmr2 are important for ATP binding and cOA synthesis, and the study further reveals a cooperative substrate binding mechanism for efficient cOA synthesis (Han et al., 2018). Cas10 with substitutions of the conserved serine residues still retains a certain degree of cOA synthesis activities, yet whether the nucleotide specificity is affected remains unclear (Han et al., 2018; You et al., 2019). It will be interesting to investigate the possibility of other cyclic oligonucleotides synthesis from uncharacterized type III effector complexes.

## cOA-Activated Effector Proteins

The effector proteins Csm6 and Csx1, representatives of CARF family proteins, can be activated by either cOA_4_ or cOA_6_, depending on their preferences (Shah et al., 2019). Very recently, the crystal structures of complexes of Csm6/Csx1 with cOA molecules have been determined (Jia et al., 2019b; Molina et al., 2019; Garcia-Doval et al., 2020). Studies on these structures reveal that one cOA binds to each CARF domains of the symmetrical homodimer of Csm6/Csx1, resulting in conformational change of Csm6/Csx1 and HEPN domain activation (Jia et al., 2019b; Molina et al., 2019; Garcia-Doval et al., 2020). Furthermore, the CARF domain of Csm6 can autoregulate its RNase activity through degrading its cOA activators (Athukoralage et al., 2019; Jia et al., 2019b; Garcia-Doval et al., 2020). However, the CARF domain of Csx1 cannot cleave its cOA activator, suggesting that the degradation of cOA by CARF domains is not a general mechanism for CARF family proteins (Molina et al., 2019).

Since CARF domain is responsible for sensing cOA, other CARF domain-containing proteins may also serve as the effector proteins. Bioinformatics analysis shows that the CARF domain is also fused to various other domains in type III systems, implying cOA signaling may provide immunity through activating various CARF domain proteins not just Csm6/Csx1 (Makarova et al., 2014, 2020a; Koonin and Makarova, 2018; Shah et al., 2019). For examples, CARF domain is fused to other RNase domains such as ribosome-dependent endoribonuclease RelE and PIN, and DNase domains of restriction endonuclease (REase) and HD nuclease, suggesting that RNA and even DNA can be degraded by such cOA-activated CARF domain proteins (Makarova et al., 2014, 2020a; Koonin and Makarova, 2018). Additionally, CARF domains are also fused with domains such as helix-turn-helix (HTH), AAA+ ATPase, or adenosine deaminase, suggesting that RNA transcription can also be regulated by such cOA-activated CARF domain proteins (Makarova et al., 2014, 2020a; Koonin and Makarova, 2018). Very recently, an effector protein containing two CARF domains and one DNA nuclease-like domain (named Can1) and another effector protein containing a Csx1 protein fused to a ring nuclease CRISPR-associated ring nuclease 2 (Crn2) domain (named Csx1-Crn2) are characterized (McMahon et al., 2020; Samolygo et al., 2020). Unlike Csm6/Csx1, Can1 is a monomeric enzyme with DNA nuclease activity (McMahon et al., 2020), while Csx1-Crn2 degrades cOA_4_ by the Crn2 domain to limit its cOA_4_-activated ribonuclease activity (Samolygo et al., 2020). These results demonstrated the diversity of CARF domain-containing effectors.

It has been known that activated Cas10 subunit produces cOAs ranging from cOA_3_ to cOA_6_ (Kazlauskiene et al., 2017). However, it is unlikely that cOA_3_ or cOA_5_ can activate CARF domain proteins like Csm6/Csx1 which assembles as homodimer with 2-fold symmetry, because the two cOAs lack symmetry to fit the dimer interface of CARF domains (Rouillon et al., 2018). Thus, it was questioned why type III effector complex generates cOA_3_ and cOA_5_, which are even the predominant products (Kazlauskiene et al., 2017; Smalakyte et al., 2020); and whether there are any other kinds of effector proteins presented in type III systems. Very recently, it is found that a novel CD-NTase produces cOA_3_, which in turn activates its effector endonuclease NucC to degrade DNA non-specifically to provide immunity against bacteriophage (Lau et al., 2020; Ye et al., 2020). Interestingly, NucC homologs as accessory proteins are also encoded within type III CRISPR/Cas systems and can be strongly activated by cOA_3_, indicating the existence of effector proteins without CARF domains in type III systems (Lau et al., 2020; Malone et al., 2020). Indeed, many other kinds of accessory proteins have been identified in type III systems (Shah et al., 2019). New cOA-activated effector proteins may still exist and remain to be identified, especially in some type III systems that contain Cas10 but lack any CARF domain proteins (Koonin and Makarova, 2018).

## Immunoprotection Conferred By cOA Signaling

The effector protein Csm6 has been shown to be essential for type III-A CRISPR-Cas systems against phage and plasmid even before the cOA signaling was discovered in 2017 (Hatoum-Aslan et al., 2014; Jiang et al., 2016). Anti-phage activity of Csm6 was demonstrated to be dependent on Cas10 activation and cOA synthesis *in vivo* at the time of cOA signaling discovery (Niewoehner et al., 2017). Since Csm6/Csx1 cleaves RNA with a preference for only one or two nucleotides (Kazlauskiene et al., 2017; Foster et al., 2019; Jia et al., 2019b; Molina et al., 2019), it is largely sequence non-specific, and degrades RNA of host and invader indiscriminately. Thus, the activated effector Csm6/Csx1 is deleterious to the host, and it was proposed that cOA signaling confers host defense through inducing cell dormancy to arrest infection or inducing programmed host cell death to abort infection (Kazlauskiene et al., 2017). Indeed, it has been observed that Csm6 activation resulted in degradation of both host and plasmid transcripts, and induced growth arrest of the host which was critical for plasmid clearance (Rostøl and Marraffini, 2019). Recently, it was found that a kind of jumbo phages form nucleus-like structures during infection to protect their DNA from DNA-targeting nucleases (Chaikeeratisak et al., 2017; Mendoza et al., 2020). However, a type III system can provide robust immunity against such nucleus-forming jumbo phage (Malone et al., 2020). In this case, the cOA signaling is essential for the type III system against the jumbo phage (Malone et al., 2020). Interestingly, the effector protein involved in the cOA signaling is a NucC-like DNA nuclease but not the ribonuclease (Malone et al., 2020), which is not capable of cleaving the jumbo phage DNA in principle. Thus, it is likely that the cOA signaling confers defense by inducing host dormancy or abortive infection through non-specifically degrading the host genome.

It is worth noting that Csm6/Csx1 activation is crucial for efficient immunity against virus when targets are late-expressed viral genes but not the early-expressed genes (Jiang et al., 2016; Bhoobalan-Chitty et al., 2019). It was suggested that the Cas10 ssDNase is sufficient to clear the invaders when targets are early-expressed genes (Jiang et al., 2016; Bhoobalan-Chitty et al., 2019). In this case, it is not necessary for the host to activate cOA signaling pathway which might also be toxic to the host. Indeed, it was shown that targeting the late-expressed viral gene exhibits a relatively stronger antiviral immunity than targeting the early-expressed viral gene when the Cas10 ssDNase is inactivated, indicating that cOA signaling-mediated immunity may be stronger in targeting the late-expressed viral gene than the early-expressed gene (Bhoobalan-Chitty et al., 2019). Recent studies have shown that the Palm domain of Cas10 can be strongly activated even when the target genes are transcribed at very low levels (Rostøl and Marraffini, 2019; Athukoralage et al., 2020b), so it is unlikely that transcripts from early-expressed genes cannot activate Cas10. One possibility is that the activity of Cas10 Palm domain is inhibited in the early infection stage by some unknown mechanism in host cells, and this inhibition is released in the late infection stage for the activation of Cas10 Palm domain. Such hypothesis could be supported by findings that cellular nucleotides such as dATP, AMP, and ADP can also bind to the adenosine binding sites of Cas10 and affect cOA synthesis (Kazlauskiene et al., 2017). The level of these nucleotides may be decreased during viral replication, promoting activation of cOA synthesis.

Histidine-aspartate domain of Cas10, which is responsible for non-specific ssDNA degradation, is also involved in immunity against viruses and plasmids. It was reported that cOA signaling should be coupled with Cas10 ssDNase activity for efficient clearance of invader genomes (Jiang et al., 2016; Rostøl and Marraffini, 2019; Varble and Marraffini, 2019). However, in some studies, inactivation of HD domain of Cas10 has little effect on immunity against invaders, suggesting that without the assistance of Cas10 ssDNase activity, the type III effector complex with cOA signaling can still provide sufficient immunoprotection (Foster et al., 2019; Liu et al., 2019; Malone et al., 2020). Cas10 ssDNase was previously proposed to be involved in ssDNA cleavage at the transcription bubble, but a recent study argued this mechanism, leaving the real role of the Cas10 ssDNase unclear (Liu et al., 2019). Thus, how cOA signaling cooperates with the Cas10 ssDNase for immune defense remains to be investigated.

## cOA Signaling Inhibition

Due to its toxicity to the host, extant cOA should be removed after clearance of the invaders to enable host cells to return to normal growth state (Figure 1B). The first cOA nuclease, also named CRISPR-associated ring nuclease 1 (Crn1), was identified from crenarchaeote *Sulfolobus solfataricus* (Athukoralage et al., 2018). Crn1 is a CARF domain-containing protein that forms a homodimer, and specifically cleaves cOA_4_ into linear di-adenylate products to switch off the cOA_4_-activated effector proteins (Athukoralage et al., 2018). Interestingly, the CARF domain of effector protein Csm6 is also found to be capable of degrading cOA, thereby functioning as self-limiting ribonucleases (Athukoralage et al., 2019; Jia et al., 2019b; Garcia-Doval et al., 2020). Notably, very recently, it was found the HEPN domain of Csm6 can also degrade cOA to self-regulate its RNase activity (Smalakyte et al., 2020). Moreover, recent studies report that the widespread CRISPR associated protein Csx3 is a novel ring nuclease, named Crn3 (CRISPR associated ring nuclease 3; Athukoralage et al., 2020a; Brown et al., 2020). Interestingly, an unusual cooperative catalytic mechanism was found in which an active site of Csx3 tetramer is formed by two dimers sandwiching a cOA_4_ substrate (Athukoralage et al., 2020a). In addition, a metal-dependent and membrane-associated DHH-DHHA1 family nuclease (MAD) from *Sulfolobus islandicus* has recently been identified as a novel cOA-degrading enzyme (Zhao et al., 2020). MAD can accelerate the clearance of high-level cOA and may cooperate with cellular ring nuclease to remove cOA (Zhao et al., 2020).

Since cOA signaling promotes strong antiviral immunity, conversely, virus can utilize different strategies to restrict cOA signaling for immune evasion. Obviously, cOA degradation is a simple and efficient way for viruses to evade immune response. Indeed, a new family of viral anti-CRISPR (Acr) protein, AcrIII-1, was recently identified as a ring nuclease that specifically degrades cOA_4_, suggesting that it functions as Acr protein against cOA_4_-triggered type III CRISPR-Cas immunity (Athukoralage et al., 2020c). AcrIII-1 has a higher activity for cOA_4_ degradation than Crn1 and Crn3, and is unrelated to the CARF family proteins (Athukoralage et al., 2020a,c). AcrIII-1 homologs are widespread in various prokaryotes, where AcrIII-1 homologs may function as host-encoded ring nuclease like Crn1 and Crn3, thus named Crn2 (Athukoralage et al., 2020c). Very recently, a novel type III CRISPR-Cas inhibitor AcrIIIB1, encoded by *Sulfolobus* virus, has been identified to inhibit type III-B system immunity by binding to its effector complex to affect cOA signaling, demonstrating another strategy developed by virus to evade cOA signaling-mediated immunity (Bhoobalan-Chitty et al., 2019).

Currently, all of the characterized cOA nucleases (Crn1–3 and MAD) specifically degrade cOA_4_. However, given that Cas10 synthesizes cOAs ranging from cOA_3_ to cOA_6_, it is rational to predict the existence of other cOA-specific nucleases in prokaryotes and viruses. A cOA_6_-activated Csm6 can cleave cOA_6_ by its CARF domain (Garcia-Doval et al., 2020), indicating there may be presence of other CARF domain proteins such as Crn1 homologs that can specifically cleave cOA_6_. Moreover, MAD which is distinct from ring nucleases has a board substrate spectrum including cyclic di-nucleotides and ssRNA, implying MAD could degrade various cOAs (Zhao et al., 2020). Additionally, AcrIIIB1 utilizes a special strategy to inhibit cOA signaling, but its homologous proteins are only found in a few archaeal viruses (Bhoobalan-Chitty et al., 2019). The inhibitory activity of AcrIIIB1 also inspires us to explore other Acr proteins in bacteriophages and more strategies for evasion of cOA signaling such as inhibiting the effector protein activities.

## Concluding Remarks

In recent studies, a large family of CD-NTases have been found to produce a wide variety of cyclic di- and trinucleotides including 3'3' cyclic UMP-AMP, 3'3'3' cyclic AMP-AMP-GMP, and 2'3'3' cyclic AMP-AMP-AMP, which had never been reported previously (Whiteley et al., 2019; Lowey et al., 2020). These cyclic di- and trinucleotides can activate the downstream effector proteins, such as patatin-like phospholipases, DNA endonucleases, proteases, and pore-forming transmembrane proteins, to mediate anti-phage immunity by abortive infection (Cohen et al., 2019; Lau et al., 2020; Lowey et al., 2020; Ye et al., 2020), which is similar to the action of effector proteins of cOA signaling in type III systems. This newly discovered anti-bacteriophage defense system is termed cyclic oligonucleotide-based anti-phage signaling system (CBASS; Cohen et al., 2019), which is widespread and diverse in bacteria and crosstalks with cOA signaling. For example, cOA_3_-activated DNA endonuclease in CBASS is also present in some type III systems and is considered to be an important effector protein of cOA signaling for immunity against phage (Lau et al., 2020; Malone et al., 2020). Very recently, it was found that the major CBASS-associated protein effectors contain a SAVED domain, which is a fusion of two CARF-like domains, but recognize diverse asymmetric cyclic oligonucleotide signals such as 3'3'3'cyclic AMP-AMP-GMP and 3'3'3' cyclic AMP-AMP-AMP (cOA_3_) which are synthesized by CD-NTases (Lowey et al., 2020). Interestingly, Bioinformatics analysis showed that CD-NTases and SAVED domains fused to protein partners such as Lon protease and pore-forming transmembrane protein, which are occasionally incorporated into type III CRISPR loci (Burroughs et al., 2015; Lowey et al., 2020), which may increase the complexity of the regulation of the cOA signaling and enhance cOA signaling-mediated immunity.

Cyclic oligonucleotide molecules discovered in the past 3 years have greatly expanded our understanding on nucleotide signaling molecules in prokaryotes for decades. Due to the diversity of the effector proteins in cOA signaling and crosstalk between cOA signaling and CBASS, cOA signaling is far from fully elucidated. Future studies of cOA regulation will further expand our understanding of the role of cOA signaling and give us new insights into the cyclic oligonucleotides involved in antiviral defense systems.

## Author Contributions

All authors listed have made a substantial, direct and intellectual contribution to the work, and approved it for publication.

## Conflict of Interest

The authors declare that the research was conducted in the absence of any commercial or financial relationships that could be construed as a potential conflict of interest.
